# Comparative Analysis of Laparoscopic Versus Open Procedures in Specific General Surgical Interventions

**DOI:** 10.7759/cureus.54433

**Published:** 2024-02-19

**Authors:** Mihir Patil, Pankaj Gharde, Kavyanjali Reddy, Krushank Nayak

**Affiliations:** 1 General Surgery, Jawaharlal Nehru Medical College, Datta Meghe Institute of Higher Education and Research, Wardha, IND

**Keywords:** surgery, endoscopy, cholecystectomy, hysterectomy, open surgery, laparoscopy

## Abstract

Laparoscopic and open surgeries are two distinct surgical approaches with significantly different procedures and outcomes. Minimally invasive surgery, also known as laparoscopic surgery, utilizes small incisions and specialized instruments like the laparoscope to perform procedures. This contrasts with open surgery, which requires larger incisions to directly access the surgical site. Open surgery was the preferred approach for any invasive procedure until the introduction of new technological advances in the form of laparoscopy. While laparoscopy is still evolving, preliminary results demonstrate promise for various operations. Open surgery provides the healthcare professional with more liberty in the form of increased visualization, but it also increases tissue damage and hospital stays. Laparoscopic and open procedures are both valuable surgical methods with advantages and disadvantages. While open surgery is favored for difficult patients, laparoscopic surgery offers a quicker recovery and fewer scars. The choice between the two approaches depends on the patient's condition, surgical demands, and the surgeon's skills. As these methods develop, they become increasingly important for offering safe and efficient surgical treatments across a range of medical specialties.

## Introduction and background

Open surgery, sometimes called conventional surgery, uses a significant incision to access underlying organs or tissues. Although minimally invasive procedures have become more popular in some circumstances, they have long been the most accepted way to perform surgery and are even being used presently [1]. The surgeon makes a cut of the required size through the skin and surrounding tissues for direct visualization and to carry out procedures. Surgeons use this incision to immediately view and work on organs or tissues that are being targeted. Open surgery is frequently carried out on patients under general anesthesia. Surgeons see the surgical site clearly and directly, which enables accurate structural identification and manipulation. Through open surgery, locations or organs that may be hard to reach using minimally invasive methods can be reached [2]. Open surgery may be recommended when the operation is complicated or involves several organs since it provides for more dexterity and control. Open surgery enables the surgeons to handle tissues directly and carry out treatments like suturing or grafting. Greater tissue damage, a longer healing period, and a higher chance of problems like infection and scarring are all possible outcomes of open surgery’s wider incisions. Compared to minimally invasive treatments, open-surgery patients frequently need a longer hospital stay. An increase in pain and suffering is possible following open surgery due to the larger incisions and tissue stress involved [3]. Compared to minimally invasive methods, open surgical recovery is typically slower. The patient may need several weeks or months to fully recuperate and get back to their regular routine [1]. Laparoscopic surgery, commonly referred to as keyhole surgery or minimally invasive surgery, uses specialized instruments and a thin, tube-shaped camera with a light source (laparoscope) to perform surgery through small incisions. Laparoscopic surgery involves using a laparoscope-a thin, tube-shaped camera with a light-and other specialized tools to perform surgery through tiny cuts. A number of tiny incisions, generally between 0.5 and 1.5 centimeters in length, are created in the abdomen wall during laparoscopic surgery. The next step is to inflate the belly with carbon dioxide gas to create a workspace and improve vision. The surgeon inserts the laparoscope into one of the incisions to see interior organs and tissues on a video monitor and other specialized instruments required for surgery via other incisions. Stress, blood loss, discomfort, scarring, infection risk, and recovery time are reduced due to smaller incisions in laparoscopic surgeries. The smaller cuts lead to less pain post-surgery and shorter hospital stays, allowing a quicker return to regular activities. The tiny incisions used during laparoscopic surgery typically lead to greater cosmetic success as well [4-6].

## Review

A comprehensive and detailed approach was used to research the topic of open surgery and laparoscopy. Results were selected from research databases worldwide, including PubMed, Google Scholar, MEDLINE, and Embase. The search terms “open vs. laparoscopic surgery,” “endoscopy,” and “laparoscopic hysterectomy” were used. We exclusively examined articles that were published in the English language. Editorials were excluded, and articles published between 2000 and the present were included. Laparoscopic surgery is frequently used for procedures such as hysterectomy, ovarian cyst removal, endometriosis treatment, and tubal ligation. Laparoscopic techniques are also being applied for differential uses such as cholecystectomy, appendectomy, hernia repair, colorectal surgery, prostate surgery, kidney surgery, treatment of urinary tract disorders, and weight loss procedures like gastric bypass and sleeve gastrectomy. These surgeries require specialized training and skills for both the surgeons and their surgical team. Laparoscopy provides less tactile feedback than open surgery, so surgeons must rely more on visual cues and instrumentation. Equipment requirements include specialized tools and equipment that are needed for laparoscopic surgery, which may not always be accessible at healthcare facilities. Certain patient factors, such as obesity, severe scarring, or certain medical conditions, may make laparoscopic surgery more difficult or less feasible [7-9].

General surgery

General surgery is a surgical specialty that treats a variety of illnesses and medical problems using surgical procedures. General surgeons are trained to perform a variety of surgical procedures on many body parts, except for the brain, spine, and heart, which are included in the purview of specialized surgical disciplines. A detailed discussion is framed herewith about general surgery, including its breadth, training, typical operations, and important practice areas. A wide variety of surgical techniques fall under the general surgery umbrella, and general surgeons are adept at handling both elective and urgent surgical patients. General surgeons perform a wide range of surgical procedures, such as abdominal surgeries, breast disorders, oncological procedures, colorectal surgeries, orthopedic surgeries, soft tissue procedures, abscess drainage, hemorrhoids, fissures, and many others. General surgeons play a vital role in treating trauma patients with chest, abdominal, and other injuries, as well as procedures for vascular diseases [10,11].

Open surgery

It is mostly referred to as "traditional surgery" or "conventional surgery," which in general contains a big incision made in the body to access and operate on underlying tissues or organs. This has the convenience of a clear view and ease of access to the surgical site with the help of conventional surgical equipment, in contrast to minimally invasive procedures that use tiny incisions and specialized devices. The patient receives general anesthesia before the surgery, ensuring there is no pain during the procedure. The region that needs surgery is exposed by the incision made by the surgeon through the skin and surrounding tissues for a particular treatment. The surgical procedure and target organs/tissues determine the size and placement of the incision. Clear site vision is attributed to this incision, which enables accurate manipulation and removal of sick or damaged tissues by providing a good view of the surgical field. Using typical surgical equipment, scalpels, sutures, and other medical tools, the surgeon carries out the required treatments, such as removing tumors, treating wounds, or reconstructing damaged organs or tissues [12]. After completion of the procedure, the surgical incision site is carefully closed by using sutures, staples, or surgical glue, ensuring that the wound is properly contained. During an open procedure, the surgeon has a clear, unobstructed view of the surgical site, which allows for exact structural identification and manipulation. Open surgery is appropriate for a variety of operations, including sophisticated and complex procedures involving many organs or systems. It makes it possible to reach places or organs that may be hard to access with minimally invasive procedures. During surgery, surgeons may directly feel and touch the tissues, which can contribute to the tactile input they get. Open surgery has been a common strategy for many years, but due to the rise in minimally invasive procedures, there is a decline in the exposure of general surgery residents to these procedures [13].

Laparoscopic surgery

Laparoscopy has seen a tremendous evolution in the field of surgery, completely altering how many surgical operations are carried out. Laparoscopy originated in the early 20th century but has undergone major advances recently, leading to widespread adoption [2]. The use of cystoscopes-devices that allow for the visualization of the bladder-to examine the abdominal cavity gave rise to the idea of laparoscopy. A modified cystoscope was used by German surgeon Georg Kelling to conduct the first laparoscopy on a dog in 1901. However, neither quick attention nor extensive usage was given to these early experiments. In 1910, Swedish surgeon Hans Christian Jacobaeus performed the first successful laparoscopy on a human. He conducted a diagnostic inspection of the abdominal cavity using a cystoscope. The word "laparoscopy" was created by Jacobaeus, who also shared his research with the medical field. The practice of expanding the abdominal cavity with gas (originally carbon dioxide), known as pneumoperitoneum, has substantially enhanced laparoscopic visibility. With the use of this method and improvements in optics, surgical results were enhanced, and images were of higher quality [14,15]. The invention of laparoscopic cholecystectomy was the innovation that propelled laparoscopy into mainstream surgery in the late 1980s. The first laparoscopic excision of the gallbladder was carried out in 1987 by German surgeon Erich Mühe. This ground-breaking technique attracted attention from all across the world by showcasing laparoscopy’s potential for significant surgical procedures [16]. Significant advancements in laparoscopic surgical methods and instruments were developed in the 1990s. High-resolution monitors and video cameras have increased visualization, while articulating equipment has given surgeons more maneuverability during surgery. After the laparoscopic cholecystectomy became successful, other surgical specialties began to use laparoscopy. Using minimally invasive methods, procedures including laparoscopic appendectomy, hernia repair, colorectal surgery, and gynecological operations became commonplace. Robot-assisted laparoscopy became more popular in the 2000s. Robotic systems like the da Vinci surgical system allowed complex operations to be done with greater precision, dexterity, and less invasion [17]. Emerging techniques in the 2000s included natural orifice transluminal endoscopic surgery (NOTES) and single-incision laparoscopic surgery (SILS). SILS minimizes the number of visible scars by doing the whole procedure through a single tiny incision, and NOTES investigates doing internal procedures without exterior incisions by entering the abdomen through natural openings, such as the mouth or anus [15]. With continual improvements in equipment, visualization, and procedures, laparoscopy is still developing. Today, a variety of laparoscopic treatments are regularly carried out by surgeons in a number of different fields, including urology, gynecology, cancer, and bariatric surgery [18,19].

Laparoscopic colorectal surgery

From being an experimental method carried out by a few pioneers in the early 1990s, laparoscopic surgery for colorectal illness is now firmly entrenched in the mainstream globally. This has happened even though laparoscopic surgery costs more and takes longer to complete than a similar open colorectal procedure. We are now entering the next phase of minimally invasive colorectal procedures. In patients with rectal cancer, laparoscopic surgery was linked to comparable rates of disease-free and overall survival, as well as locoregional recurrence and less blood loss as compared to open surgery. Large-scale multicenter randomized trial data have established that laparoscopic colorectal surgery is safe both in terms of short-term perioperative outcomes and long-term oncological efficacy [14,20-21].

Laparoscopic cholecystectomy

Laparoscopic cholecystectomy is a minimally invasive removal of the gallbladder. This procedure provides a less-invasive alternative to traditional open gallbladder removal surgery. In cases of gallstones, cholecystitis, or other gallbladder conditions, laparoscopic cholecystectomy is a minimally invasive procedure for the removal of the gallbladder. The abdomen is cut at different sites using a number of tiny incisions, typically between 0.5 and 1.5 centimeters long, for the entry of laparoscopic tools. In contrast, open cholecystectomy is surgery to remove the gallbladder through a large abdominal incision. To remove a gallbladder during an open cholecystectomy, the surgeon creates a long incision in the upper right abdomen, just below the ribs. This incision is normally 15 to 20 centimeters long. This method of gallbladder removal was the standard approach before the advent of laparoscopic cholecystectomy [16,19,22-23]. Carbon dioxide gas is injected into the abdominal cavity to create a working area. Surgeons can maneuver the tools and see the operative site well because of this inflation. Insertion of the laparoscope is done through a small cut, which also allows the insertion of other laparoscopic tools such as a camera and light source. The surgery site is viewed in real-time due to the transmission of high-definition images to the video monitor by the inserted camera. These images provide a magnified picture of the surgical region and are used by the surgeon to guide their actions. Other specialized laparoscopic tools are inserted through the other incisions. During the process, these tools are utilized to cut, suture, and manipulate tissues. The gallbladder is carefully separated from its liver and bile duct connections before being extracted. Operating surgeons keep a check on the bleeding and surrounding tissues to be unharmed during and after the removal of the gallbladder, followed by the incisions being stitched or sealed with medical glue. There is minimal scarring due to small incisions, which patients find attractive from an aesthetic standpoint. Patients who have laparoscopic surgery often suffer fewer postoperative discomforts and a shorter recovery time as compared to patients who underwent regular open cholecystectomy [19]. Compared to open surgery, laparoscopic cholecystectomy is frequently conducted as an outpatient treatment. The choice to have a laparoscopic cholecystectomy is dependent on the patient’s particular medical history and general state of health; thus, it is crucial for the surgeon to evaluate each case individually to choose the best surgical course [22]. While laparoscopic cholecystectomy has become the preferred method for most gallbladder removals due to its minimally invasive nature, open cholecystectomy is still performed in certain cases where laparoscopic surgery is not feasible or safe. The method of surgery utilized to remove the gallbladder is the main distinction between open and laparoscopic cholecystectomy. Laparoscopic surgery is less invasive, and there is often less chance of infection and other issues afterward. Compared to open surgery, patients can resume normal activities faster after laparoscopic surgery with a shorter hospital stay [19,22,23].

Rectus diastasis

The choice between open versus laparoscopic rectus diastasis surgery is heavily affected by the degree of rectus diastasis, the patient’s general health, the experience of the surgeon, and the patient's preferences. Laparoscopic rectus diastasis surgery is a newer procedure, and enough research evidence on its long-term outcomes is not available. However, studies have shown that it is safe and effective in relieving symptoms and improving the appearance of the abdomen. If the patient qualifies for less invasive techniques due to the severity of the diastasis, laparoscopic rectus diastasis surgery is typically selected. On the contrary, if the patient has severe diastasis or is in a particular clinical condition where laparoscopic surgery is difficult or the risk is high, open surgical methods are opted for. The divided abdominal muscles are accessed and repaired during open surgery through a single, significant abdominal incision. The rectus abdominis muscles are directly in reach of the surgeon for repair through the incision, which is commonly performed along the midline of the abdomen as compared to laparoscopic surgery, where there are some small incisions through which this whole procedure is performed. The surgery site is viewed with the help of a laparoscope and other specialized instruments, which are required to be placed through other incisions for closing rectus diastasis [24-27].

Laparoscopic hysterectomy

A hysterectomy is carried out for different benign clinical conditions. It is approached by both methods, viz., open surgery through the abdomen and laparoscopic surgeries. As evidenced by the available research literature around the world, wider incisions and resulting tissue stress in open abdominal hysterectomy required a longer recovery period, while patients undergoing laparoscopic hysterectomy surgery recovered in less time on account of smaller incisions, less blood loss, and less tissue stress [28]. The first laparoscopic hysterectomy procedure was described and carried out successfully in 1989, with the authors reporting a shorter recovery period and suggesting using laparoscopic hysterectomy in selective cases to decrease morbidity and recovery period [29,30].

Laparoscopic hernia repair

Hernia repair has been attempted in both open and laparoscopic ways. Meta-analysis reports have reported that both methods had nearly similar chances of recurrence; the surgery time and length of hospitalization in both methods were nearly similar. Research evidence has reported higher incidences of recurrence rates in laparoscopic procedures as compared to open surgical procedures, but the meta-analyses based on their results mentioned that the recurrences in both open and laparoscopic methods are equivalent [31,32] with research evidence of compared outcomes of laparoscopic and robotic surgery [33].

Laparoscopic appendectomy

Laparoscopic appendectomy has been a preferred choice over open surgery in the management of acute appendicitis. There are reasons like a lower number of wounds, a shorter hospitalization period, and a need for a lesser amount of analgesics listed most commonly for this preference. An umbrella review of ten meta-analyses mentioned 48-70% fewer surgical site infections (RR: 0.56 (0.47-0.67)) but a higher risk of intra-abdominal abscess (1.20 (0.88-1.63)) as compared to open surgeries [34]. Laparoscopic versus open surgery for the management of acute appendicitis has also been researched in pregnant women in a systematic review and meta-analysis, as laparoscopic procedures have been given a preference over open surgery options [35]. This study concluded that there was an insignificant association between pre-term delivery and laparoscopic and open surgical methods for the management of acute appendicitis in pregnancy. Overall, laparoscopic appendectomy procedures can be considered for minimal morbidity, short hospital stays, lower infection risk, reduced re-admissions, and higher success rates [34-37].

Open versus laparoscopic surgery

Open surgery has been a common practice for many years, and the majority of surgeons are skilled in this method. The advantage of open surgery is direct visualization. Open surgery gives the surgeon a direct, unobstructed view of the surgical site, which can be helpful in cases with complicated or difficult anatomical structures [17]. The major drawback of open surgery is the large incision, which is its major benefit. This incision is large and results in more tissue injury, longer hospital stays, and a slow-paced recovery. Laparoscopic surgery has an edge over open surgeries, which include minimally invasive procedures that afflict minimal tissue injury, which further reduces the hospital stay period and can induce a faster recovery [2,3,36].

Limitations

Major limitations associated with laparoscopic surgery include a lack of technical expertise, as it requires extensive training to perform laparoscopic surgery and advanced, sophisticated tools. Laparoscopic surgery relies mostly on visual cues and tools, offering less touch input than open surgery. Also, laparoscopic surgery options may be strenuous and not available to all patients due to medical conditions limiting their universal applicability, unlike open surgeries, which are performed in limited resource settings with the same clinical outcomes [11,17,18]. In circumstances where laparoscopic surgery is unsafe and not possible due to severe inflammation, significant scarring, bleeding issues, or other complicating reasons, open surgery may be chosen. There are benefits and drawbacks to both open surgery and laparoscopic surgery and what may be ‘better’ for one patient may not be the same for another [37,38].

## Conclusions

It is difficult to declare a definitive winner in this comparison between open surgery and laparoscopic surgery when assessing which method is superior. The choice of which strategy to employ depends on the condition and unique circumstances of the patient to be operated on. Both approaches have their own set of benefits and drawbacks. To determine the optimal surgical approach to ensuring both patient safety and the best results, a thorough assessment of the patient’s medical history, clinical suitability, and operating personnel skills is necessary. Laparoscopic methods have been developed and mastered for some surgical interventions based on minimally invasive procedures that are capable of reducing in-hospital stays, postoperative complications, costs, and recovery periods.
